# Identification of a small mutation panel of coding sequences to predict the efficacy of immunotherapy for lung adenocarcinoma

**DOI:** 10.1186/s12967-019-02199-6

**Published:** 2020-01-14

**Authors:** Ying Li, Wenbin Jiang, Tianhao Li, Mengyue Li, Xin Li, Zheyang Zhang, Sainan Zhang, Yixin Liu, Wenyuan Zhao, Yunyan Gu, Lishuang Qi, Lu Ao, Zheng Guo

**Affiliations:** 1 College of Bioinformatics Science and Technology, Harbin Medical University, Harbin, 150086 China; 2 Department of Bioinformatics, Key Laboratory of Ministry of Education for Gastrointestinal Cancer, School of Basic Medical Sciences, Fujian Medical University, Fuzhou, 350001 China; 3 Key Laboratory of Medical Bioinformatics, Fujian Province, Fuzhou, 350001 China

**Keywords:** Immunotherapy, Tumour mutation burden, CDS mutation panel, Lung adenocarcinoma, Clinical application

## Abstract

**Background:**

Immune checkpoint inhibitors are effective in some cases of lung adenocarcinoma (LUAD). Whole-exome sequencing has revealed that the tumour mutation burden (TMB) is associated with clinical benefits among patients from immune checkpoint inhibitors. Several commercial mutation panels have been developed for estimating the TMB regardless of the cancer type. However, different cancer types have different mutational landscapes; hence, this study aimed to develop a small cancer-type-specific mutation panel for high-accuracy estimation of the TMB of LUAD patients.

**Methods:**

We developed a small cancer-type-specific mutation panel based on coding sequences (CDSs) rather than genes, for LUAD patients. Using somatic CDSs mutation data from 486 LUAD patients in The Cancer Genome Atlas (TCGA) database, we pre-selected a set of CDSs with mutation states significantly correlated with the TMB, from which we selected a CDS mutation panel with a panel-score most significantly correlated with the TMB, using a genetic algorithm.

**Results:**

A mutation panel containing 106 CDSs of 100 genes with only 0.34 Mb was developed, whose length was much shorter than current commercial mutation panels of 0.80–0.92 Mb. The correlation of this panel with the TMB was validated in two independent LUAD datasets with progression-free survival data for patients treated with nivolumab plus ipilimumab and pembrolizumab immunotherapies, respectively. In both test datasets, survival analyses revealed that patients with a high TMB predicted via the 106-CDS mutation panel with a cut-point of 6.20 mutations per megabase, median panel score in the training dataset, had a significantly longer progression-free survival than those with a low predicted TMB (log-rank *p* = 0.0018, HR = 3.35, 95% CI 1.51–7.42; log-rank *p* = 0.0020, HR = 5.06, 95% CI 1.63–15.69). This small panel better predicted the efficacy of immunotherapy than current commercial mutation panels.

**Conclusions:**

The small-CDS mutation panel of only 0.34 Mb is superior to current commercial mutation panels and can better predict the efficacy of immunotherapy for LUAD patients, and its low cost and time-intensiveness make it more suitable for clinical applications.

## Background

Lung adenocarcinoma (LUAD) is the most common type of non-small cell lung cancer (NSCLC), accounting for approximately 80–90% cases of lung cancer [1]. Currently, approximately 35–75% of LUAD patients relapse or die within 5 years of receiving conventional treatments based on the National Comprehensive Cancer Network Clinical Practice Guidelines in Oncology [2]. Recently, immunotherapies, which eliminate tumours by activating the immune system [3], have shown great promise for NSCLC [4, 5]. For example, an immune checkpoint inhibitor, nivolumab, which targets programmed cell death protein-1 (PD-1), can significantly increase survival in advanced-stage NSCLC by blocking the interaction between PD-1 and its ligand programmed death-ligand 1 (PD-L1) and allowing cytotoxic T lymphocytes to act on tumour cells [6]. Furthermore, the inhibitor (ipilimumab) for cytotoxic T lymphocyte-associated antigen 4 (CTLA-4), which suppresses immune responses, has been approved for treating NSCLC [5] and some other cancers [7]. However, the heterogeneity of the response to immune checkpoint inhibitors significantly confounds the treatment of NSCLC [3]. Therefore, it is important to identify patients potentially benefiting from these immune checkpoint inhibitors.

Previously, PD-L1 protein expression in NSCLC patients has been approved as an auxiliary predictive marker for certain PD-1/PD-L1 inhibitors including pembrolizumab [8]. However, PD-L1 protein expression alone cannot completely account for the survival benefit to patients treated with immune checkpoint inhibitors [8–11]. Moreover, analysis of PD-L1 protein expression via immunohistochemistry (IHC) is challenging because of subjective diagnoses of immunostaining results by pathologists using different criteria or interpretations [12].

Several previous studies have reported a high tumour mutation burden (TMB), determined through whole-exome sequencing (WES), indicating that patients are more likely to harbour neoantigens, can predict the sensitivity to immunotherapies [13, 14]. For example, high-TMB patients are associated with enhanced responses to nivolumab (PD-1 inhibitor) plus ipilimumab (CTLA-4 inhibitor) immunotherapy [15]. Moreover, a high TMB is more significantly associated with the response to PD-1/PD-L1 inhibitors than with PD-L1 protein expression detected via IHC [16]. However, WES, necessary to determine the TMB, is not routinely performed in clinical practice because it is costly, time-consuming and labour intensive, and needs a large number of sequences [3, 17, 18]. Previous studies have reported that the TMB can be accurately estimated using smaller gene panels encompassing several hundred genes, such as the 324-gene mutation panel (FoundationOne CDxTM assay) [6, 19–21] and the 341-gene mutation panel (MSK-IMPACT) [22, 23], which have been clinically used. The cost-effectiveness of these mutation panels facilitates a greater sequencing depth than that of WES and consequently a higher ability to detect mutations, even for genes mutated in some tumour cells [24]. However, these commercial mutation panels were selected from cancer-related genes regardless of the cancer type, rather than being developed via a feature selection method; thus, mutation panels can still be improved. In particular, it is necessary to develop a cancer-type-specific mutation panel to estimate the TMB of LUAD samples, since different cancer types have different mutation landscapes [25]. Recently, Lyu et al. [3] constructed a LUAD-specific 24-gene model for predicting the TMB of LUAD samples. However, this panel was also based on complete exons of the panel genes, comprising thousands of exons in the panel genes, most of which being unmutated, solely increasing the unnecessary cost and time for sequencing.

In this study, based on the coding sequences (CDSs) with a high frequency of mutation in LUAD, we developed a CDS mutation panel to estimate the TMB of LUAD samples. Thereafter, we determined the correlation of CDSs in the mutation panel with the TMB in two independent datasets. From two datasets (Matthew and Rizvi), we used data on progression-free survival (PFS) of advanced LUAD patients treated with immune checkpoint inhibitors to estimate the performance of the CDS mutation panel for predicting the efficacy of immunotherapy. Furthermore, the CDS mutation panel was compared with two commercial mutation panels (324-gene and 341-gene panels) and a LUAD-specific mutation panel (24-gene panel).

## Methods

### Data sources and pre-processing

The three LUAD somatic mutation datasets (Table 1) were used to construct and validate the mutation panel for estimating the TMB. The training mutation data were downloaded from The Cancer Genome Atlas (TCGA) database (https://portal.gdc.cancer.gov/), comprising 486 LUAD samples with paired mRNA expression data. For validation, we obtained two independent somatic mutation datasets with PFS data for patients treated with immune checkpoint inhibitors, including 59 LUAD samples reported by Matthew et al. [5] and 29 LUAD samples reported by Rizvi et al. [4]. The patients included in the Matthew dataset were treated with nivolumab (PD-1 inhibitor) plus ipilimumab (CTLA-4 inhibitor) and those in the Rizvi dataset were treated with pembrolizumab (PD-1 inhibitor) immunotherapy.

**Table 1:** Whole-exome sequencing mutation data analyzed in this study

Patient characteristics	TCGA	Matthew [5]	Rizvi [4]
	No. (%)	No. (%)	No. (%)
Histology			
Adenocarcinoma	486	59	29
Age (years)			
No less than 65	223 (46)	29 (50)	10 (34)
Less than 65	263 (54)	30 (50)	19 (66)
Sex			
Male	222 (46)	22 (37)	13 (45)
Female	264 (54)	37 (63)	16 (55)
Smoking status			
Never	–	13 (22)	5 (17)
Former/light	–	38 (64)	18 (62)
Current/heavy	–	8 (14)	6 (21)
Stage			
I	263 (54)	–	–
II	117 (24)	–	–
IIIA	70 (14)	–	–
IIIB–IV	36 (7)	59 (100)	29 (100)
PFS-status			
Progression	–	40 (68)	20 (69)
Progression-free	–	19 (32)	9 (31)
Percentage of tumour cells			
Known	433 (89)	–	–
Unknown	53 (11)	–	–
Average percentage of tumour cells	78.76	–	–

Whole-exome sequencing was previously performed for these TCGA data with tumour tissues and matched normal tissue or blood, which were used to filter out germline mutations and screen somatic mutations [26]; the detailed protocol is described in the original literature [27]. Briefly, 0.5–3 µg of DNA from each sample was used for library preparation and sequenced using the Illumina HiSeq platform. The mean coverage across targeted bases on tumour and germline DNA were 97.63 and 95.83, respectively. Mutations with a variant allelic fraction of < 0.05 in tumour tissue were excluded. Only the non-synonymous mutations, including missense mutation, nonsense mutation, nonstop mutation, frame-shift mutation and in-frame mutation, were included, and a discrete mutation profile including 82,574 CDSs of 16,961 genes was generated. For the two test mutation data, whole-exome sequencing was performed for tumour tissues and matched normal tissues or blood. The detailed protocol is further described in the original literatures [5, 28]. Finally, discrete mutation profiles including 18,793 CDSs of 9400 genes and 8711 CDSs of 5504 genes were generated, wherein the CDSs mutation matrix was constructed using matched human reference genome annotated files derived from GENCODE (https://www.gencodegenes.org/human/releases.html).

### Development of the CDS mutation panel for estimating TMB

First, from TCGA LUAD somatic mutation data, we extracted mutations in the CDSs using the human reference genome annotated file (GRCh38), and selected non-synonymous mutations to construct an m*n CDSs mutation matrix, where *m* represents the number of CDSs in genes and *n* represents the number of samples. TMB was estimated as (total mutations in CDSs/total bases of CDSs) * 10^6^.

Thereafter, Spearman’s rank correlation analysis was performed to estimate the correlation of the CDSs mutation state with the TMB. Herein, we restricted the analysis to the CDSs mutated in more than 5% cancer samples [29, 30] to filter out ‘passenger’ genes with low-frequency mutations, as it may be subjected to random mutations rather than having a tumorigenic advantage. p-values were adjusted using the Benjamini–Hochberg procedure [31] for multiple testing to control the false discovery rate (FDR). CDSs significantly correlated with the TMB were selected as candidates.

Finally, the genetic algorithm (GA package) was used to generate a final CDS panel from among candidate CDSs, whose panel-score was most correlated with TMB. The genetic algorithm was implemented with a population size of 5000 and a crossover fraction of 0.9; it was terminated if the optimization objective of the best subset was not improved in 100 generations. Details regarding the genetic algorithm are shown in Additional file 1. The correlation (R^2^) was estimated via linear regression analysis [32]. Here, the panel-score was calculated as following (Formula 1):

1 $${\text{Panel-score}} = \beta \frac{{\sum\nolimits_{i = 1}^{n} {k_{i} } }}{{l*10^{{{ - }6}} }} + C$$

where *n* is the number of CDSs in the panel, *l* is the length of the panel, and $k_{i}$ is the number of mutations in *i*-th CDS; $\beta$ and $C$ was obtained through linear regression analysis, $\beta$ is a coefficient to balance the panel-score and TMB, $C$ is a constant.

As no clinical data regarding immunotherapy were available for patients in TCGA, we could not determine the optimal cut-point for our CDS panel for predicting the efficacy of immunotherapy. Therefore, we set the cut-point of our CDS panel at a median panel score in TCGA.

### Survival analysis

PFS was defined as the period during and after the treatment of a disease, wherein a patient lives with the disease but it is not exacerbated. The survival curve was estimated using the Kaplan–Meier method and compared using the log-rank test (survival package: ‘survdiff’) [33]. The univariate Cox proportional hazards regression model (survival package: ‘coxph’) was used to evaluate the predictive performances of the mutation panels. Furthermore, the multivariate Cox model (survival package: ‘coxph’) was used to evaluate the independent prognostic value of our CDS mutation panel after adjusting for clinical factors including age, sex, and smoking. Hazard ratios (HRs) and 95% confidence intervals (CIs) were generated using the Cox proportional hazards model (survival package: ‘coxph’).

### Functional enrichment analysis

Functional pathways for enrichment analysis were downloaded from Gene Ontology (GO) in November 2018. First, we performed Student’s t-test with a 5% FDR control to select differentially expressed genes (DE genes) between the high-TMB and low-TMB groups classified by the CDS panel. Here, 17,680 genes were used for differential expression analysis. Thereafter, the hypergeometric distribution model was used to determine whether the number of DE genes observed in a functional term was significantly greater than that expected through random chance.

All statistical analyses were performed by using R software packages version 3.4.2 (http://www.r-project.org/). Significance was defined as *p* < 0.05 or FDR < 0.05 for multiple testing.

## Results

### Construction of the CDS mutation panel

**Fig. 1:**
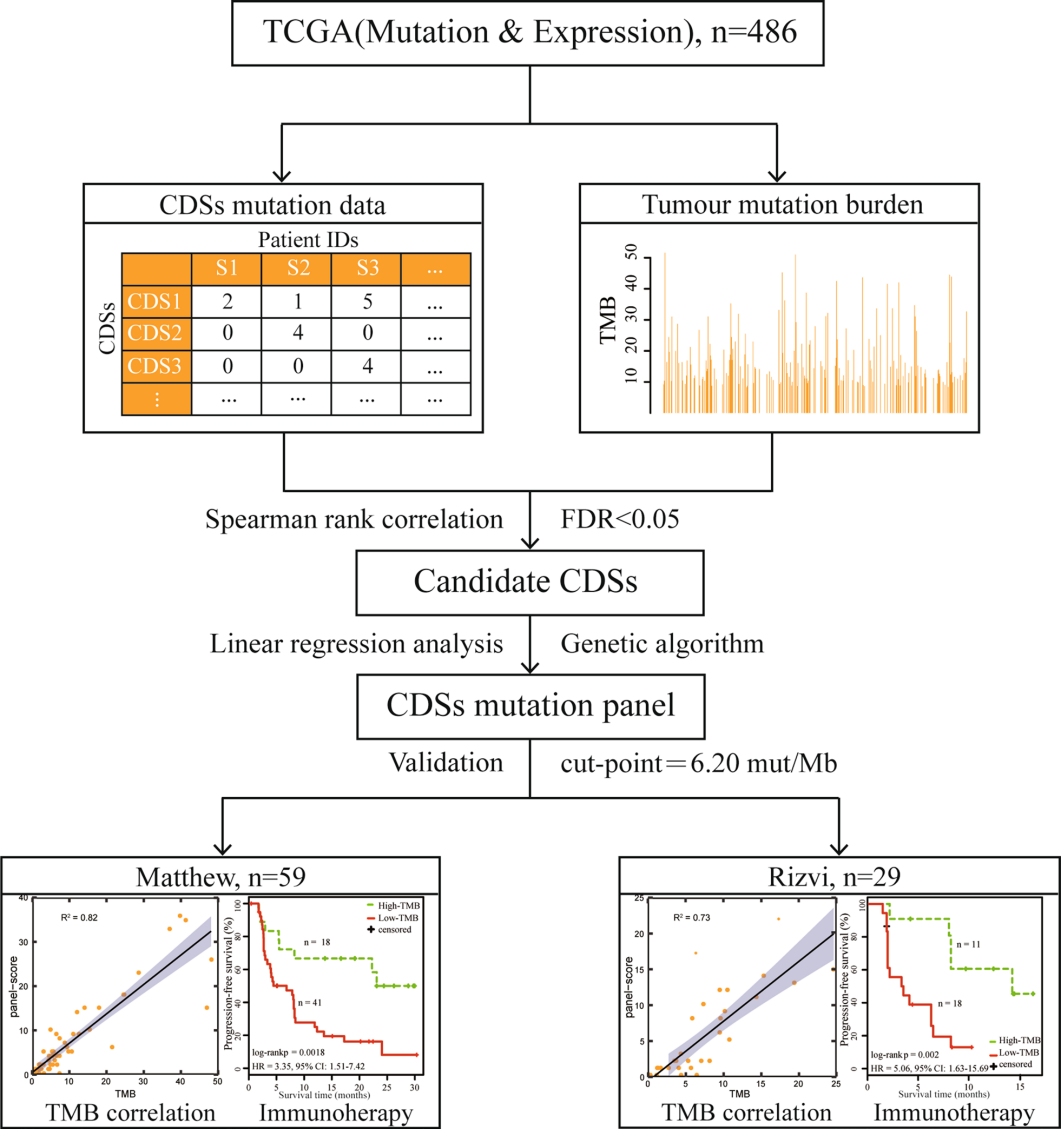
Flowchart for the identification of the mutation panel. Mutation data for coding sequences (CDSs) in TCGA from 486 patients was used to develop a CDS mutation panel for estimating the tumour mutation burden. The performance of the CDS mutation panel was validated in two datasets with data on progression-free survival for patients treated with immune checkpoint inhibitors

Figure 1 provides a schematic representation of the study protocol.

**Fig. 2:**
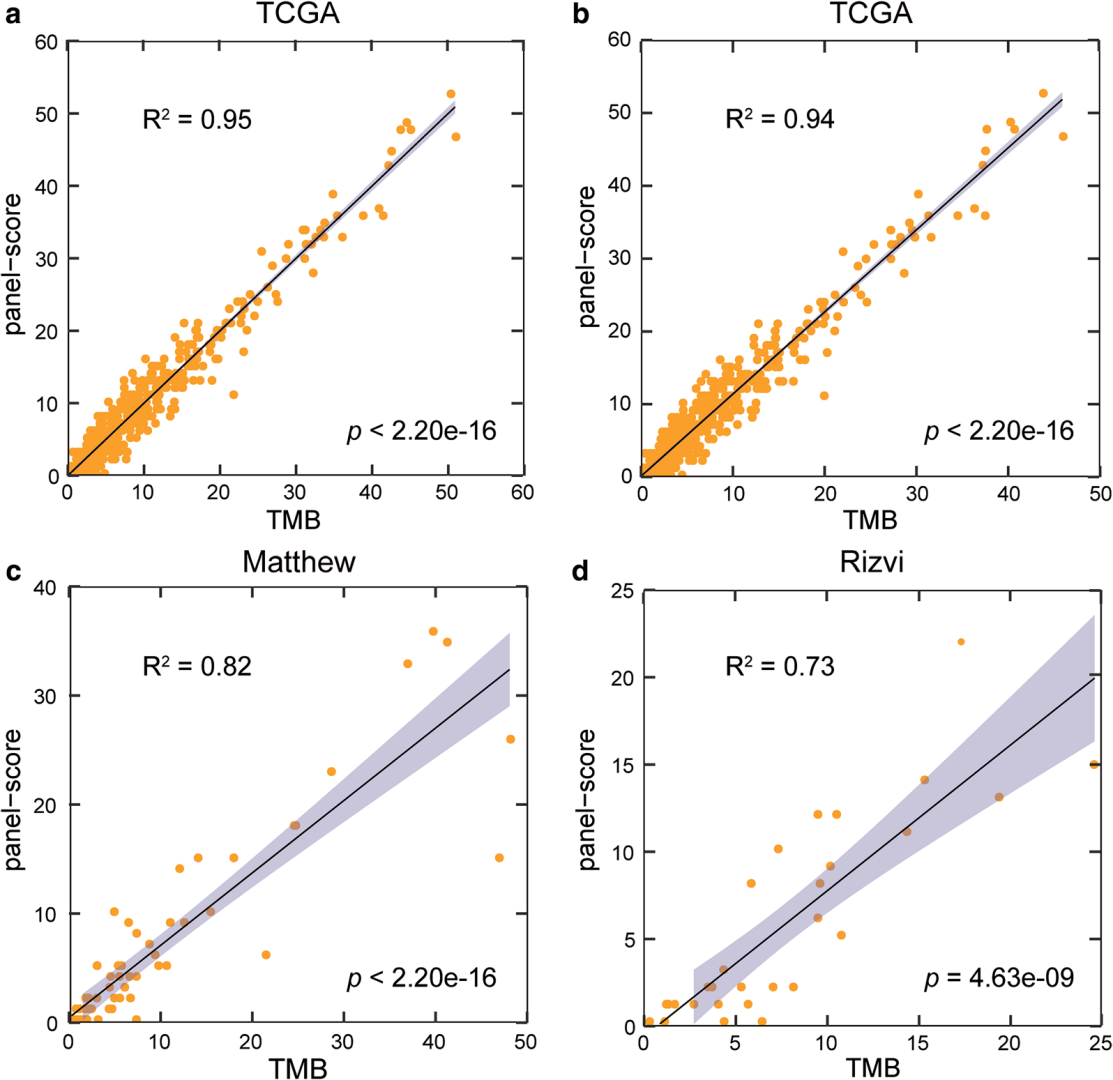
Performance of the 106-CDS panel for the tumour mutation burden evaluation. **a** Linear regression analysis of the 106-CDS panel-score with the tumour mutation burden (TMB) of lung adenocarcinoma (LUAD) in TCGA database (training set). **b** Linear regression analysis of the 106-CDS panel-score with the recalculated TMB in TCGA after excluding the mutations in cancer-related genes and the recurrent CDSs occurring in more than 5% samples. **c** Linear regression analysis of the 106-CDS panel-score and the TMB in the Matthew dataset. **d** Linear regression analysis of the 106-CDS panel-score and the TMB in the Rizvi dataset. The gray lines are 95% confidence intervals of the 106-CDS panel for the TMB evaluation

From the LUAD mutation data from TCGA, we extracted mutation data from the CDSs by using the human reference genome annotated file (GRCh38). After selecting non-synonymous mutations, a mutation matrix comprising 82,574 CDSs and 486 patients was generated. Thereafter, using Spearman’s rank correlation analysis, with a 5% FDR control, 219 CDSs were significantly correlated with the TMB of the LUAD samples derived from TCGA data. Using the genetic algorithm (“Methods”), we extracted a CDS panel comprising 106 CDSs in 100 genes with a length of 0.34 Mb (Additional file 2: Table S1), whose panel-score was most significantly correlated with the TMB of the LUAD samples (R^2^ = 0.95, Fig. 2a). This mutation panel was termed the 106-CDS panel. In the formula for the panel-score, $\beta = 0.33$, ${\text{C = 0}} . 2 7$ were obtained from linear regression analysis [32]. The technical details of the 106-CDS panel for TMB evaluation is described in Additional file 3: Table S2.

Furthermore, we estimated the correlation of the clinical factors and tumour cell proportions with the TMB using Spearman’s rank correlation and found that only age and sex were significantly associated with the TMB (age: *p* = 0.0055; sex: *p* = 0.0442, Additional file 4: Table S3). Therefore, we additionally used the multiple linear regression model for the 106-CDS panel, age and sex together to estimate their correlations with the TMB. Consequently, the 106-CDS panel was still significantly correlated with the TMB, while age and sex were not, suggesting that the 106-CDS panel was an independent predictor of the TMB. Additionally, to prevent overestimating the TMB, since gene panels are usually heavily targeted at recurrently mutated genomic regions, we redetermined the TMB after excluding the mutations in cancer-related genes and the recurrent CDSs occurring in more than 5% samples, and found that the correlation (R^2^) of the 106-CDS panel with the recalculated TMB also approached 0.94 (Fig. 2b).

### Validation of the 106-CDS panel

**Fig. 3:**
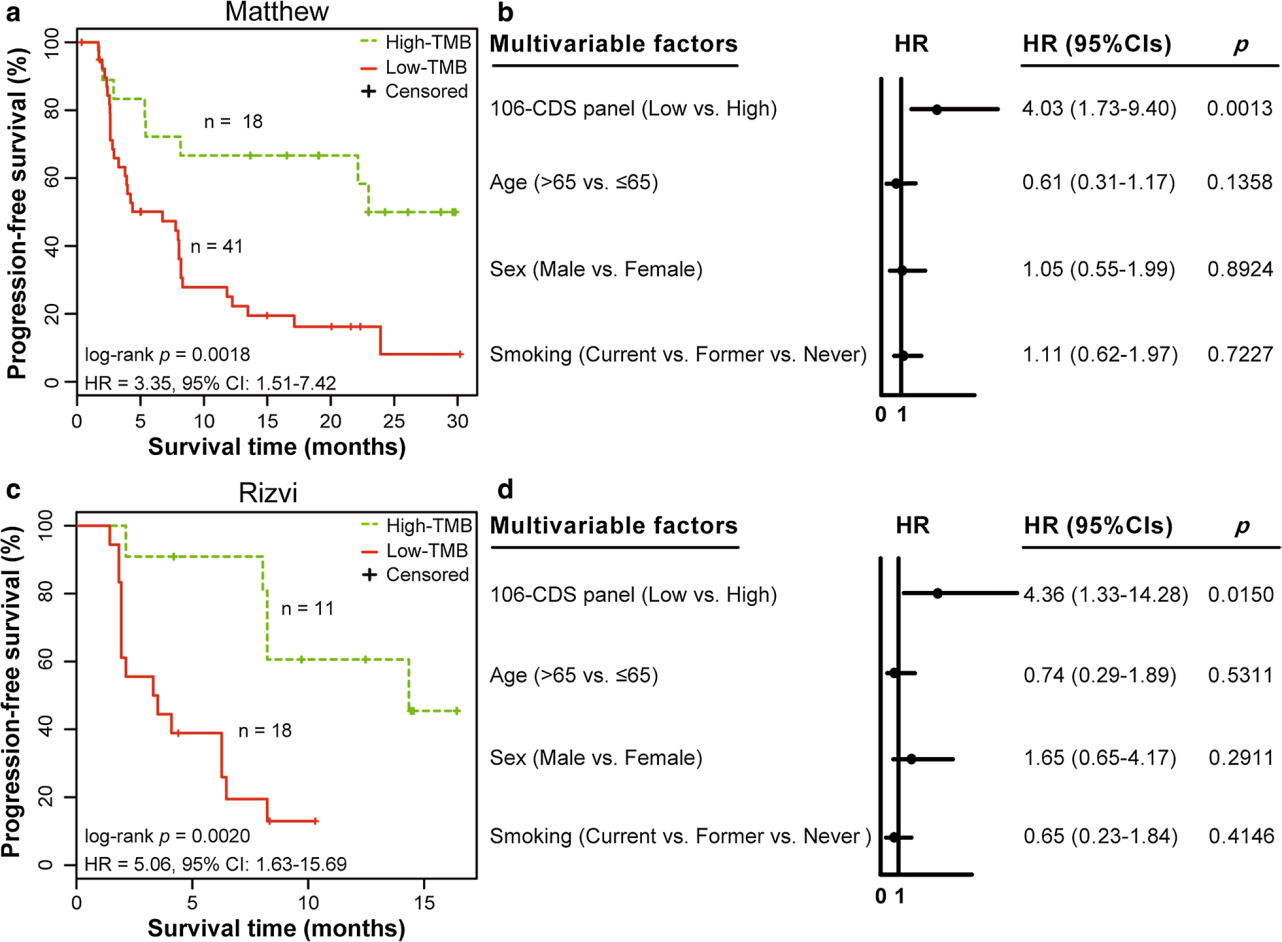
Performance of the 106-CDS panel for predicting the efficacy of immunotherapy in the test datasets. **a** Kaplan–Meier curves of progression-free survival (PFS) for the 59 advanced lung adenocarcinoma (LUAD) patients in the Matthew dataset. The *p* value was determined using log-rank test. The hazard ratio (HR) and 95% confidence interval (CI) were determined using univariate Cox regression models. **b** Multivariate Cox analysis for the 106-CDS panel, age, sex, and smoking status in the Matthew dataset. Solid circles represent the HR for mortality risk and the open-ended horizontal lines represent the 95% CI. The *p* value, HR, and CI were determined using multivariate Cox regression models. **c** Kaplan–Meier curves of PFS for the 29 advanced LUAD patients in the Rizvi dataset. The *p* value was determined using log-rank test. The HR and 95% CI were determined using univariate Cox regression models. **d** Multivariate Cox analysis of the 106-CDS panel, age, sex, and smoking status in the Rizvi dataset. The *p* value, HR, and CI were determined using multivariate Cox regression models

First, we applied the 106-CDS panel to 59 samples from the Matthew dataset with PFS data of patients receiving nivolumab plus ipilimumab immunotherapy. The correlation (R^2^) between the panel-score of the 106-CDS and the TMB was 0.82 (linear regression analysis, *p* < 0.0001, Fig. 2c). When the panel score was dichotomized at 6.20 mutations per megabase (mut/Mb), the median of the panel-scores from the training dataset, our 106-CDS panel classified 18 and 41 patients into high- and low-risk groups, respectively. Univariate survival analysis revealed that the predicted high-TMB patients had significantly longer PFS than the predicted low-TMB patients (log-rank *p* = 0.0018, HR = 3.35, 95% CI 1.51–7.42, Fig. 3a). The 1-year PFS rate of the predicted high-TMB patients was 0.67, which was markedly higher than the 1-year PFS rate (0.25) of the predicted low-TMB patients. Multivariate Cox analysis revealed that the 106-CDS panel with a cut-point of 6.20 mut/Mb remained significantly associated with PFS (*p* = 0.0013, HR = 4.03, 95% CI 1.73–9.40, Fig. 3b) after adjusting for age (> 65 vs. ≤ 65 years), sex (Male vs. Female), and smoking status (Current vs. Former vs. Never).

Similar results were obtained with the Rizvi dataset, wherein the correlation (R^2^) between panel-score and the TMB was 0.73 (linear regression analysis, *p* < 0.0001, Fig. 2d). High-TMB patients predicted using the 106-CDS panel with a cut-point of 6.20 mut/Mb had a significantly longer PFS than the predicted low-TMB patients (log-rank *p* = 0.0020, HR = 5.06, 95% CI 1.63–15.69, Fig. 3c). The 1-year PFS rate of the predicted high-TMB patients was 0.61, markedly higher than the 1-year PFS rate (0.13) of the predicted low-TMB samples. Multivariate Cox analysis revealed that the CDS panel with a cut-point of 6.20 mut/Mb remained significantly associated with PFS (*p* = 0.0150, HR = 4.36, 95% CI 1.33–14.28, Fig. 3d) after adjusting for age (> 65 vs. ≤ 65 years), sex (male vs. female), and smoking status (current vs. former vs. never).

### Comparison of the 106-CDS panel with three mutation panels

We compared our 106-CDS panel with two commercial mutation panels (324-gene [6, 20, 21] and 341-gene [23] panels) approved by the Food and Drug Administration (FDA), and a LUAD-specific mutation panel (24-gene panel) for predicting the efficacy of immunotherapy for LUAD. The results of univariate survival analyses are summarized in Table 2.

**Table 2:** The survival analysis result of all datasets

Dataset	Mutation panels	Log-rank *p*	Cox *p*^a^	Hazard ratio (95% CIs)^a^
Matthew	106-CDS	0.0018	0.0029	3.35 (1.51–7.42)
	324-gene	0.0042	0.0057	2.65 (1.33–5.28)
	341-gene	0.0135	0.0156	2.20 (1.16–4.17)
	24-gene	0.0283	0.0312	2.04 (1.07–3.89)
Rizvi	106-CDS	0.0020	0.0050	5.06 (1.63–15.69)
	324-gene	0.0137	0.0208	3.74 (1.22–11.46)
	341-gene	0.1233	0.1193	2.06 (0.83–5.14)

^a^Cox *p* value and Hazard ratio (95% CIs) were generated by the univariate Cox proportional hazards model

**Fig. 4:**
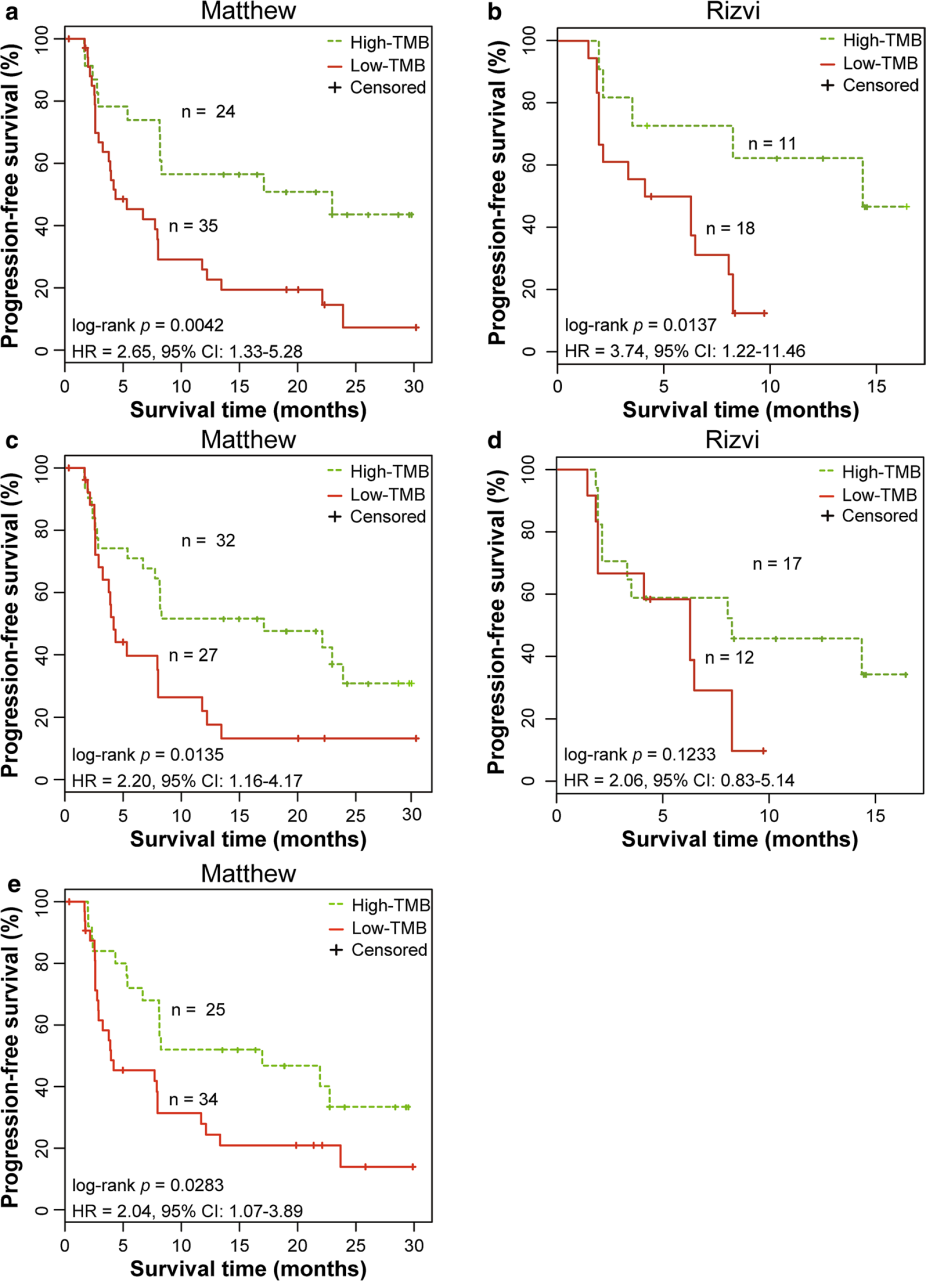
Performance of other mutation panels for predicting the efficacy of immunotherapy in the test datasets. **a** Kaplan–Meier curves of progression-free survival (PFS) for 59 advanced lung adenocarcinoma (LUAD) patients in the Matthew dataset, using the 324-gene panel. **b** Kaplan–Meier curves of PFS for 29 advanced LUAD patients in the Rizvi dataset, using the 324-gene panel. **c** Kaplan–Meier curves of PFS for 59 patients in the Matthew dataset, using the 341-gene panel. **d** Kaplan–Meier curves of PFS for 29 patients in the Rizvi dataset, using the 341-gene panel. **e** Kaplan–Meier curves of PFS for 59 patients in the Matthew dataset, using the LUAD-specific 24-gene panel. The *p* value was determined using log-rank test. The hazard ratio (HR) and 95% confidence interval (CI) were determined using univariate Cox regression models

For the 324-gene mutation panel with a cut-point of 10 mut/Mb [19], containing 6130 CDSs spanning 0.80 Mb, univariate survival analyses revealed that the two groups of patients classified using the panel had significantly different PFS after receiving immunotherapy in the Matthew dataset (log-rank p = 0.0042, HR = 2.65, 95% CI 1.33–5.28, Fig. 4a) and in the Rizvi dataset (log-rank p = 0.0137, HR = 3.74, 95% CI 1.22–11.46, Fig. 4b). However, univariate survival results revealed that its performance (HR) in predicting the efficacy of immunotherapy was worse than that of our 106-CDS panel in both datasets (Table 2).

For the 341-gene mutation panel with a cut-point of 7.40 mut/Mb [22], containing 6773 CDSs spanning 0.92 Mb, its performance in predicting the efficacy of immunotherapy in the Matthew dataset (log-rank *p* = 0.0135, HR = 2.20, 95% CI 1.16–4.17, Fig. 4c) and the Rizvi dataset (log-rank *p* = 0.1233, HR = 2.06, 95% CI 0.83–5.14, Fig. 4d) was inferior to that of our 106-CDS panel (Table 2).

For the LUAD-specific mutation panel (24-gene panel) with a cut-point of 141 [3], containing 833 CDSs spanning 0.18 Mb, univariate survival analyses revealed that the two groups of patients classified by the panel had significantly different PFS after immunotherapy in the Matthew dataset (log-rank *p* = 0.0283, HR = 2.04, 95% CI 1.07–3.89, Fig. 4e). However, its performance (HR) was much worse than that of our 106-CDS panel (Table 2). As the Rizvi dataset is the training set to determine the cut-point (141) of the 24-gene panel in predicting the benefits of pembrolizumab immunotherapy, we did not compare our 106-CDS panel with the 24-gene panel in the dataset, as it is not an independent test data for the 24-gene panel.

### Functional characterizations of the 106-CDS panel

**Fig. 5:**
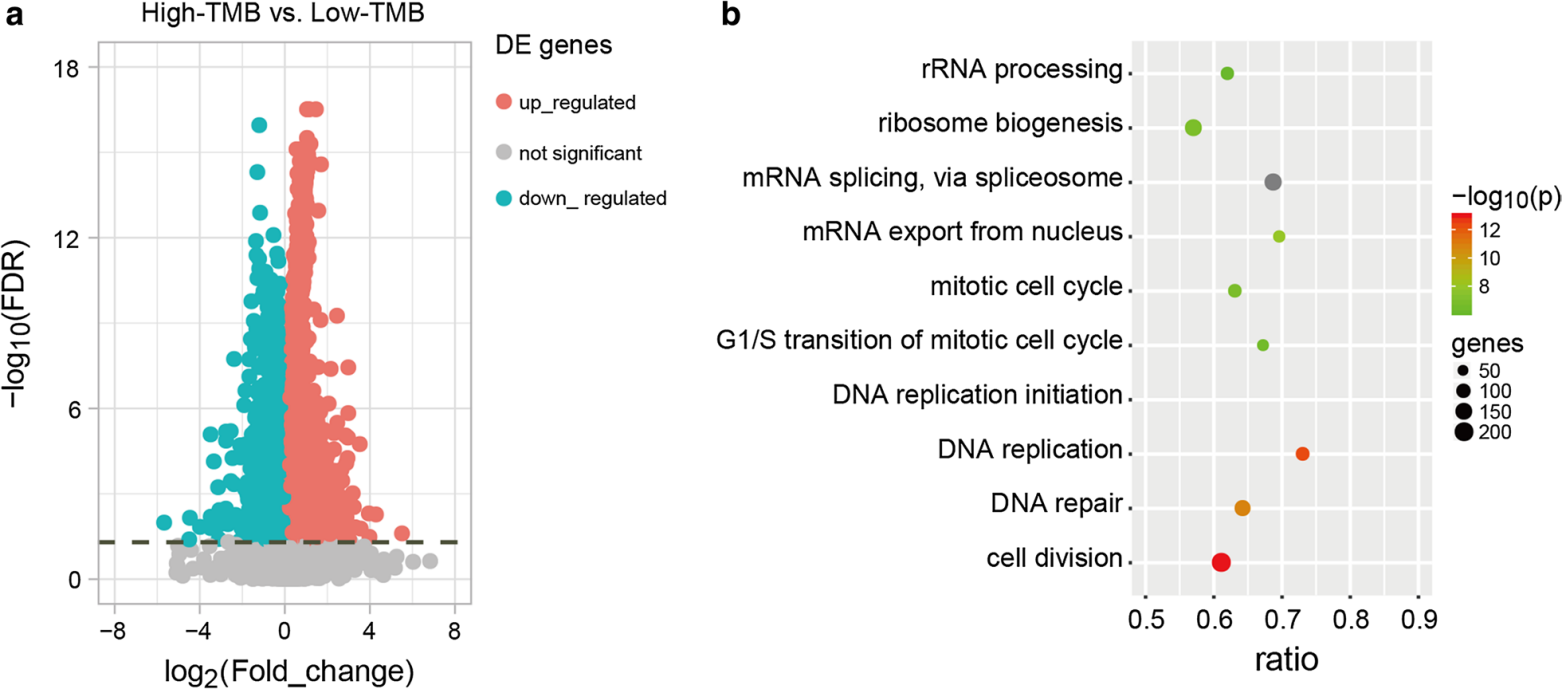
Functional characterizations of the 106-CDS panel. **a** Volcano plot of differently expressed genes (DE genes) between the high-TMB and low-TMB groups predicted via the 106-CDS panel. The list of DE genes is shown in Additional file 5: Table S4. The pink and blue circles represent the up-regulated and down-regulated DE genes in the predicted high-TMB group when compared with the predicted low-TMB group. The gray circle represent the genes without different expression between the predicted high-TMB and low-TMB groups. **b** The top 10 functional pathways significantly enriched with DE genes between the high-TMB and low-TMB groups predicted via the 106-CDS panel. All 22 functional pathways are shown in Additional file 6: Table S5. The size of nodes represents the number of DE genes in the pathway. The colour of the nodes, from green to red, represents the *p*-value of enrichment results from high to low. The ratios represent the proportion of DE genes enriched in the pathway to the total number of genes in the pathway

In TCGA dataset, using the 106-CDS panel with a cut-point of 6.20 mut/Mb, 220 and 266 samples were divided into high- and low-TMB groups, respectively. We found that 7181 genes were differentially expressed between the two groups (Student’s t-test, FDR < 0.05, Fig. 5a, Additional file 5: Table S4), which were significantly enriched in 22 functional pathways (hypergeometric distribution model, FDR < 0.05, Fig. 5b, Additional file 6: Table S5), including those associated with genomic instability, such as DNA repair [34], DNA replication [35] and chromosome segregation [36]. These results indicated that compared with the low-TMB patients predicted using the 106-CDS panel, the predicted high-TMB patients might have higher genomic instability, thus potentially benefiting from immunotherapy, as they are more likely to harbour neoantigens.

## Discussion

This study describes the generation of a mutation panel comprising 106 CDSs of 100 genes spanning 0.34 Mb. Previous studies have reported that the sequencing panel, comprising more than 300 cancer-related genes, can help predict the TMB; however, its performance is apparently low when the number of genes in the panel is lesser than 150 [37]. However, these commercial mutation panels (such as 324-gene and 341-gene panels) were not selected through any feature selection method; thus, their high correlations with the TMB primarily resulted from the large number of genes included in the panels. In contrast, our 106-CDS mutation panel developed using a genetic algorithm and containing more major variates associated with the TMB is expected to be reliable in estimating the TMB, and its performance was validated in the two independent test datasets. Certain differences in the correlations of our 106-CDS panel and the TMB were observed in the two test datasets, thus potentially accounting for their different sample sizes or sample collections; these correlations require further validation in a large-scale clinical trial.

The present results show that the 106-CDS panel with a cut-point of 6.20 mut/Mb preferably predicted the efficacy of immunotherapy among advanced-stage LUAD patients. For high-TMB patients predicted via the 106-CDS panel with a cut-point of 6.20 mut/Mb, immunotherapy with nivolumab plus ipilimumab improved the 1-year PFS rate to 0.67, which was markedly higher than the 1-year PFS rate (0.25) of the predicted low-TMB patients. Similarly, the 1-year PFS rate of the predicted high-TMB patients was 0.61, being markedly higher than the 1-year PFS rate (0.13) of the predicted low-TMB patients after pembrolizumab treatment. However, we considered that the cut-point of the 106-CDS panel, which was set at a median panel score in training dataset, may not be the optimal threshold for predicting the efficacy of various immunotherapy drugs. In order to assess the effect of specific cut-points for predicting the efficacy of immunotherapy, we additionally set the cut-points of our CDS panel at upper tertiles (9.17 mut/Mb) and quartiles (12.13 mut/Mb) of panel scores in training dataset, respectively, and estimated in the two test datasets. The univariate survival analyses revealed that the 106-CDS panel with the cut-point of the upper quartiles (12.13 mut/Mb) had the optimal predictive performance (log-rank *p* = 0.0079, HR = 3.81, 95% CI 1.33–10.93, Additional file 7: Figure S1A) than the median (log-rank *p* = 0.0018, HR = 3.35, 95% CI 1.51–7.42, Fig. 3a) and upper tertiles (log-rank *p* = 0.0298, HR = 2.59, 95% CI 1.07–6.27, Additional file 7: Figure S1B) as cut-pionts for the patients treated with nivolumab plus ipilimumab in the Matthew dataset. While, it had the weakest performance (log-rank *p* = 0.1258, HR = 2.58, 95% CI 0.72–9.21, Additional file 7: Figure S1C) than the median (log-rank *p* = 0.0020, HR = 5.06, 95% CI 1.63–15.69, Fig. 3c) and upper tertiles (log-rank *p* = 0.0081, HR = 5.82, 95% CI 1.33–25.51, Additional file 7: Figure S1D) for the patients treated with pembrolizumab in the Rizvi dataset. These results suggest that the 106-CDS panel with a cut-point of 6.20 mut/Mb can effectively predict patients potentially benefiting from immunotherapies, but the optimal cut-point for a specific immunotherapy drug needs further exploration in a large-scale clinical trial.

The larger the number of genes included in the mutation panel, the higher the expected correlation with the TMB. Our results show that although the number of genes in the 106-CDS panel is twofold less than that of the 324-gene [19] and 341-gene [22] panels, our 106-CDS panel displayed better performance in predicting the efficacy of immunotherapy. Although the length of the 106-CDS panel (0.34 Mb) was longer than the 24-gene panel (0.18 Mb), its performance was markedly better in predicting the efficacy of immunotherapy. These results indicate that the 106-CDS panel of mutations may have higher antigenicity, which needs further confirmation.

Functional annotation revealed that several genes including TP53 [38], AMER1 [39], and TEX15 [40] in the 106-CDS panel are involved in DNA repair and cell cycle arrest, playing a key role in genomic instability. DE genes between the two groups classified using the 106-CDS panel with a cut-point of 6.20 mut/Mb were significantly enriched in several pathways associated with genomic instability, such as DNA repair [34], DNA replication [35], and chromosome segregation [36]. These functional analyses indicate that compared with low-TMB patients predicted using the 106-CDS panel, the high-TMB patients potentially have higher genomic instability and are more likely to harbour neoantigens.

## Conclusions

The CDS mutation panel spanning only 0.34 Mb can effectively predict the efficacy of immunotherapy for LUAD patients through accurate estimation of the TMB. This small panel is preferable for clinical samples because of its low cost and time consumption.

## Supplementary information

**Additional file 1.** The genetic algorithm for searching a CDS panel.

**Additional file 2: Table S1.** Description of the 106-CDS panel.

**Additional file 3: Table S2.** Technical details of TMB evaluation.

**Additional file 4: Table S3.** The correlation of the clinical factors and tumour cell proportions with the tumour mutation burden.

**Additional file 5: Table S4.** List of differently expressed genes between high-TMB and low-TMB groups predicted via the 106-CDS panel.

**Additional file 6: Table S5.** The functional pathways enriched with differently expressed genes.

**Additional file 7: Figure S1. ** Performance of 106-CDS panel with the other cut-points for predicting the efficacy of immunotherapy.

## Data Availability

The datasets of this article were generated from the TCGA database and two articles published by Rizvi et al. [4] and Matthew et al. [5].

## Abbreviations

LUAD: lung adenocarcinomas
NSCLC: non-small cell lung cancer
PD-1: programmed death-1
PD-L1: programmed death-ligand 1
CTLA-4: cytotoxic T lymphocyte-associated antigen 4
IHC: immunohistochemistry
TMB: tumour mutation burden
WES: whole-exome sequencing
CDSs: coding sequences
PFS: progression-free survival
TCGA: The Cancer Genome Atlas
FDR: false discovery rate
HRs: hazard ratios
CIs: confidence intervals
GO: Gene Ontology
DE genes: differentially expressed genes
FDA: Food and Drug Adminstration
