# Supplementary material for: Identification of a small mutation panel of coding sequences to predict the efficacy of immunotherapy for lung adenocarcinoma
Source: J Transl Med. 2020 Jan 14;18:25. doi: 10.1186/s12967-019-02199-6 (PMC6961230; doi:10.1186/s12967-019-02199-6)
Supplement: Supplementary file 1 — Additional file 1. The genetic algorithm for searching a CDS panel. [file 12967_2019_2199_MOESM1_ESM.docx]

**The genetic algorithm for searching a CDSs panel**

We used the genetic algorithm (GA package) [1] to search the final feature subset that resulted in the largest product of the linear regression correlation with TMB. The genetic algorithm is given by the following pseudocode: Input: feature subsets fs, population size ps = 5,000, candidate feature set, optimal object o = linear regression correlation (R^2^), maximum number of generations mg = 5,000s, maximum unimproved generations mug = 100, mutation rate mr = 0.1, crossover rate cr = 0.8. Algorithm detail: (1) Generate a population of feature subsets fs and population size ps from the candidate feature set at random. (2) Evaluate the fitness of each feature subset using the optimal object, o. (3) According to the roulette selection method, the higher the fitness, the higher probability be selected, and the two feature subsets are selected from the population as parents. (4) The chromosomes (feature subsets) of both parents are extracted, and cross-interchange is performed according to the crossover rate to generate progeny. (5) The chromosome of the progeny is point-mutated according to the mutation rate, that is, a "0" or "1" of a random site on the subset has a certain probability to become "1" or "0" opposite thereto. (6) Repeat steps 3, 4, and 5 until the new population is produced. (7) When the number of iterations reaches the preset maximum number of generations mg, or exceeds the maximum unimproved generations mug, the loop is terminated. Output: p, the population of feature subsets sorted by optimal object fitness.

**References**

1. Service MW. Sequel to Kitzmiller’s Anopheline Names: Their Derivations and Histories. J Vector Ecol. 2010;35:213–66.
