# Supplementary material for: Identification of a small mutation panel of coding sequences to predict the efficacy of immunotherapy for lung adenocarcinoma
Source: J Transl Med. 2020 Jan 14;18:25. doi: 10.1186/s12967-019-02199-6 (PMC6961230; doi:10.1186/s12967-019-02199-6)
Supplement: Supplementary file 2 — Additional file 2: Table S1. Description of the 106-CDS panel. [file 12967_2019_2199_MOESM2_ESM.docx]

**Table S1.** Description of the 106-CDS panel

| **Index** | **Gene symbol** | **Initiation** | **Termination** | **Length(bp)** |
| --- | --- | --- | --- | --- |
| 1 | TCHH | 152107388 | 152113078 | 5691 |
| 2 | HRNR | 152213079 | 152221490 | 8412 |
| 3 | DUSP27 | 167125787 | 167128605 | 2819 |
| 4 | BRINP3 | 190098021 | 190099134 | 1114 |
| 5 | ASPM | 197100431 | 197105185 | 4755 |
| 6 | OR2G2 | 247588360 | 247589310 | 951 |
| 7 | OR2W3 | 247895587 | 247896528 | 942 |
| 8 | OR2AK2 | 247965332 | 247966336 | 1005 |
| 9 | OR2L13 | 248099376 | 248100311 | 936 |
| 10 | OR2M5 | 248145148 | 248146083 | 936 |
| 11 | OR2T33 | 248272855 | 248273814 | 960 |
| 12 | OR2T12 | 248294619 | 248295578 | 960 |
| 13 | OR2G6 | 248521647 | 248522594 | 948 |
| 14 | OR2T34 | 248573804 | 248574757 | 954 |
| 15 | LRRC7 | 70038113 | 70039793 | 1681 |
| 16 | LPPR4 | 99305685 | 99307007 | 1323 |
| 17 | GPR158 | 25597772 | 25599271 | 1500 |
| 18 | RBP3 | 47348485 | 47351538 | 3054 |
| 19 | PCDH15 | 53821861 | 53823358 | 1498 |
| 20 | MUC5B | 1240851 | 1251743 | 10893 |
| 21 | KCNA4 | 30010720 | 30012678 | 1959 |
| 22 | LRRC4C | 40114373 | 40116292 | 1920 |
| 23 | OR52R1 | 4803436 | 4804380 | 945 |
| 24 | OR4A5 | 54706885 | 54707829 | 945 |
| 25 | OR4A15 | 55367884 | 55368915 | 1032 |
| 26 | OR4C15 | 55554307 | 55555416 | 1110 |
| 27 | OR5D13 | 55773438 | 55774379 | 942 |
| 28 | OR5L2 | 55827219 | 55828151 | 933 |
| 29 | OR8J3 | 56136774 | 56137718 | 945 |
| 30 | OR8K3 | 56318307 | 56319242 | 936 |
| 31 | OR5R1 | 56417261 | 56418232 | 972 |
| 32 | OR1S1 | 58214745 | 58215719 | 975 |
| 33 | AHNAK | 62516747 | 62534074 | 17328 |
| 34 | NLRP10 | 7959647 | 7961322 | 1676 |
| 35 | FAT3 | 92797836 | 92801909 | 4074 |
| 36 | FZD10 | 130162943 | 130164685 | 1743 |
| 37 | PCDH20 | 61411246 | 61413966 | 2721 |
| 38 | LRFN5 | 41886626 | 41888010 | 1385 |
| 39 | FSCB | 44504513 | 44506987 | 2475 |
| 40 | UNC13C | 54012904 | 54015886 | 2983 |
| 41 | SALL1 | 51138688 | 51141930 | 3243 |
| 42 | CMTR2 | 71283611 | 71285920 | 2310 |
| 43 | TP53 | 7674181 | 7674290 | 110 |
| 44 | TP53 | 7675053 | 7675215 | 163 |
| 45 | TP53 | 7675053 | 7675238 | 186 |
| 46 | ZNF521 | 25224345 | 25227697 | 3353 |
| 47 | ZNF208 | 21971194 | 21974807 | 3614 |
| 48 | ZNF536 | 30443563 | 30445732 | 2170 |
| 49 | ZNF536 | 30547943 | 30549514 | 1572 |
| 50 | NLRP5 | 56026913 | 56028509 | 1597 |
| 51 | PEG3 | 56813678 | 56817579 | 3902 |
| 52 | USP29 | 57128676 | 57131441 | 2766 |
| 53 | MUC16 | 8934888 | 8939567 | 4680 |
| 54 | MUC16 | 8945497 | 8967189 | 21693 |
| 55 | AMER3 | 130762073 | 130764655 | 2583 |
| 56 | XIRP2 | 167242569 | 167251947 | 9379 |
| 57 | TTN | 178559311 | 178576416 | 17106 |
| 58 | TTN | 178745588 | 178752039 | 6452 |
| 59 | MAP2 | 209692637 | 209696350 | 3714 |
| 60 | APOB | 21005080 | 21012651 | 7572 |
| 61 | SPHKAP | 228016406 | 228020156 | 3751 |
| 62 | SLC8A1 | 40428473 | 40430271 | 1799 |
| 63 | ZNF831 | 59191020 | 59194757 | 3738 |
| 64 | OR11H1 | 15528159 | 15529136 | 978 |
| 65 | PCDH10 | 133150141 | 133152771 | 2631 |
| 66 | PCDH10 | 133150141 | 133152828 | 2688 |
| 67 | BOD1L1 | 13598946 | 13605084 | 6139 |
| 68 | KIAA1211 | 56314040 | 56316689 | 2650 |
| 69 | PDHA2 | 95840151 | 95841314 | 1164 |
| 70 | PCDHA2 | 140794965 | 140797436 | 2472 |
| 71 | PCDHA4 | 140807188 | 140809572 | 2385 |
| 72 | PCDHB2 | 141094791 | 141097184 | 2394 |
| 73 | PCDHB4 | 141121999 | 141124383 | 2385 |
| 74 | PCDHB5 | 141135435 | 141137819 | 2385 |
| 75 | PCDHB7 | 141172836 | 141175214 | 2379 |
| 76 | PCDHB8 | 141178035 | 141180437 | 2403 |
| 77 | PCDHB16 | 141182560 | 141184887 | 2328 |
| 78 | PCDHB9 | 141187319 | 141189709 | 2391 |
| 79 | PCDHB12 | 141208908 | 141211292 | 2385 |
| 80 | PCDHB14 | 141223506 | 141225899 | 2394 |
| 81 | PCDHGA5 | 141364331 | 141366751 | 2421 |
| 82 | CMYA5 | 79728915 | 79739403 | 10489 |
| 83 | TMEM200A | 130440423 | 130441895 | 1473 |
| 84 | PPP1R3A | 113877726 | 113880125 | 2400 |
| 85 | ABCA13 | 48271787 | 48276565 | 4779 |
| 86 | POM121L12 | 53035672 | 53036559 | 888 |
| 87 | ZNF804B | 89333363 | 89337029 | 3667 |
| 88 | ZFPM2 | 105801047 | 105803535 | 2489 |
| 89 | FAM135B | 138151194 | 138153216 | 2023 |
| 90 | TEX15 | 30842004 | 30849017 | 7014 |
| 91 | KCNB2 | 72935935 | 72938088 | 2154 |
| 92 | ZFHX4 | 76704089 | 76706678 | 2590 |
| 93 | TAF1L | 32630102 | 32635579 | 5478 |
| 94 | SPATA31A3 | 66986457 | 66990189 | 3733 |
| 95 | PRUNE2 | 76704761 | 76710237 | 5477 |
| 96 | SPATA31E1 | 87884913 | 87888822 | 3910 |
| 97 | RGAG1 | 110450618 | 110454664 | 4047 |
| 98 | DCAF12L2 | 126164536 | 126165924 | 1389 |
| 99 | DCAF12L1 | 126551220 | 126552608 | 1389 |
| 100 | GPR112 | 136344392 | 136350433 | 6042 |
| 101 | SLITRK2 | 145822426 | 145824597 | 2172 |
| 102 | FAM47A | 34129906 | 34132278 | 2373 |
| 103 | FAM47B | 34942832 | 34944766 | 1935 |
| 104 | FAM47C | 37008411 | 37011515 | 3105 |
| 105 | AMER1 | 64189882 | 64193286 | 3405 |
| 106 | TBX22 | 80030498 | 80031108 | 611 |

Note: Initiation represents the starting position of the CDSs on the chromosome; Termination represents the termination position of the CDSs on the chromosome
