# Supplementary material for: Identification of a small mutation panel of coding sequences to predict the efficacy of immunotherapy for lung adenocarcinoma
Source: J Transl Med. 2020 Jan 14;18:25. doi: 10.1186/s12967-019-02199-6 (PMC6961230; doi:10.1186/s12967-019-02199-6)
Supplement: Supplementary file 3 — Additional file 3: Table S2. Technical details of TMB evaluation. [file 12967_2019_2199_MOESM3_ESM.docx]

**Table S2.** Technical details of TMB evaluation

| Type | Parameter |
| --- | --- |
| Cancer type | LUAD |
| Input material | FFPE or frozen |
| Tumor cellularity | >60% cellularity, <20% necrosis |
| DNA extraction | DNEasy (Qiagen) |
| Capture | SureSelect All Exon v5 (50Mbp) (Agilent) |
| Depth of coverage | 97.63× |
| Sequencer | HiSeq 2000 (Illumina) |
| Mutation types | nonsyn. |
| Filtering germline | (Blood or normal tissue) |
| Ref. | [1] |

**Reference**

1. Network CGAR. Comprehensive molecular profiling of lung adenocarcinoma. Nature. 2014/07/09. 2014;511:543–50.
