# Supplementary material for: Identification of a small mutation panel of coding sequences to predict the efficacy of immunotherapy for lung adenocarcinoma
Source: J Transl Med. 2020 Jan 14;18:25. doi: 10.1186/s12967-019-02199-6 (PMC6961230; doi:10.1186/s12967-019-02199-6)
Supplement: Supplementary file 4 — Additional file 4: Table S3. The correlation of the clinical factors and tumour cell proportions with the tumour mutation burden. [file 12967_2019_2199_MOESM4_ESM.docx]

**Table S3.** The correlation of the clinical factors and tumour cell proportions with the tumour mutation burden

| **Patient Characteristics** | **TCGA** | **Matthew[5]** | **Rizvi[4]** |
| --- | --- | --- | --- |
|  | *p** | *p** | *p** |
| Age (>65 vs. ≤65) | 0.0055 | 0.5388 | 0.4319 |
| Sex (Male vs. female) | 0.0442 | 0.3635 | 0.7165 |
| Smoking status (Current vs. Former vs. Never) | - | 0.8809 | 0.8903 |
| Stage (IV vs. III vs. II vs. I) | 0.6426 | - | - |
| Percentage of tumour cells | 0.3629 | - | - |

Note: **p* value was estimated using spearman rank correlation method
