# Supplementary material for: Identification of a small mutation panel of coding sequences to predict the efficacy of immunotherapy for lung adenocarcinoma
Source: J Transl Med. 2020 Jan 14;18:25. doi: 10.1186/s12967-019-02199-6 (PMC6961230; doi:10.1186/s12967-019-02199-6)
Supplement: Supplementary file 6 — Additional file 6: Table S5. The functional pathways enriched with differently expressed genes. [file 12967_2019_2199_MOESM6_ESM.docx]

**Table S5.** The functional pathways enriched with differently expressed genes

| **GO pathways** | **Path.G** | **D**.**G** | **p** |
| --- | --- | --- | --- |
| mRNA splicing, via spliceosome | 231 | 158 | <0.0001 |
| cell division | 339 | 206 | <0.0001 |
| DNA replication | 125 | 91 | <0.0001 |
| DNA repair | 208 | 133 | <0.0001 |
| mRNA export from nucleus | 98 | 68 | <0.0001 |
| DNA replication initiation | 26 | 24 | <0.0001 |
| ribosome biogenesis | 268 | 152 | <0.0001 |
| mitotic cell cycle | 140 | 88 | <0.0001 |
| G1/S transition of mitotic cell cycle | 94 | 63 | <0.0001 |
| rRNA processing | 136 | 84 | <0.0001 |
| regulation of transcription involved in G1/S transition of mitotic cell cycle | 22 | 20 | <0.0001 |
| mitotic sister chromatid segregation | 31 | 25 | <0.0001 |
| G2/M transition of mitotic cell cycle | 123 | 74 | <0.0001 |
| viral process | 338 | 177 | <0.0001 |
| mRNA cis splicing, via spliceosome | 22 | 19 | <0.0001 |
| chromosome segregation | 64 | 43 | <0.0001 |
| RNA metabolic process | 44 | 32 | <0.0001 |
| mRNA 3-end processing | 55 | 38 | <0.0001 |
| termination of RNA polymerase II transcription | 34 | 26 | <0.0001 |
| regulation of G2/M transition of mitotic cell cycle | 80 | 51 | <0.0001 |
| CENP-A containing nucleosome assembly | 42 | 30 | <0.0001 |
| ciliary basal body-plasma membrane docking | 95 | 58 | <0.0001 |

Note: Path.G: Genes in the GO pathway; D.G, DE genes in the GO pathway
