# Supplementary material for: Identification of a small mutation panel of coding sequences to predict the efficacy of immunotherapy for lung adenocarcinoma
Source: J Transl Med. 2020 Jan 14;18:25. doi: 10.1186/s12967-019-02199-6 (PMC6961230; doi:10.1186/s12967-019-02199-6)
Supplement: Supplementary file 7 — Additional file 7: Figure S1. Performance of 106-CDS panel with the other cut-points for predicting the efficacy of immunotherapy. [file 12967_2019_2199_MOESM7_ESM.docx]

**Figure S1.** Performance of 106-CDS panel with the other cut-points for predicting the efficacy of immunotherapy.


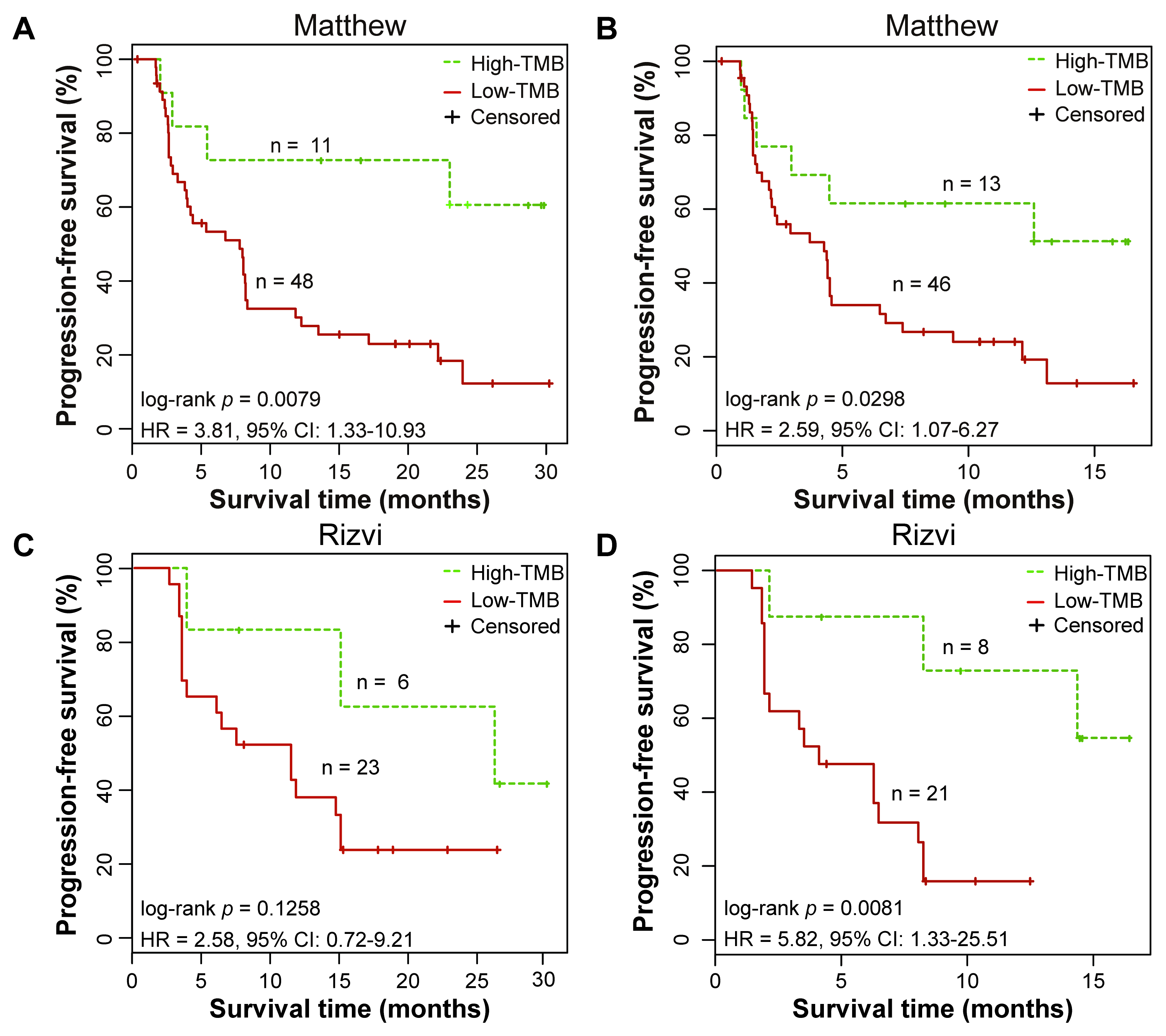


**Figure S1.** Performance of 106-CDS panel with the other cut-points for predicting the efficacy of immunotherapy. a Kaplan–Meier curves of progression-free survival (PFS) for 59 advanced lung adenocarcinoma (LUAD) patients in the Matthew dataset, using the 106-CDS panel with the cut-point of the upper quartiles. b Kaplan–Meier curves of progression-free survival (PFS) for 59 advanced lung adenocarcinoma (LUAD) patients in the Matthew dataset, using the 106-CDS panel with the cut-point of the upper tertiles. c Kaplan–Meier curves of progression-free survival (PFS) for 29 advanced lung adenocarcinoma (LUAD) patients in the Rizvi dataset, using the 106-CDS panel with the cut-point of the upper quartiles. d Kaplan–Meier curves of progression-free survival (PFS) for 29 advanced lung adenocarcinoma (LUAD) patients in the Rizvi dataset, using the 106-CDS panel with the cut-point of the upper tertiles. The hazard ratio (HR) and 95% confidence interval (CI) were determined using univariate Cox regression models
